# Optimizing Small Local Language Models for Culturally Competent Mental Health Counseling: Comparative Evaluation With GPT-4o by Psychiatrists

**DOI:** 10.2196/92470

**Published:** 2026-10-22

**Authors:** Jaewook Han, Beomgi So, Whanbo Jung, Minwoo Kim, Hoon Kim, Daun Shin

**Affiliations:** 1 Department of Psychiatry Seoul National University College of Medicine Seoul Republic of Korea; 2 Drpresso Inc Seoul Republic of Korea; 3 Department of Psychiatry Asan Medical Center University of Ulsan College of Medicine Seoul Republic of Korea

**Keywords:** large language models, small language models, cultural competence, natural language processing, digital mental health

## Abstract

**Background:**

Large language models (LLMs) show promise for mental health counseling applications. However, their deployment faces significant challenges, including data privacy concerns requiring on-premise solutions, high operational costs, and Western-centric biases that limit cultural appropriateness for non–English-speaking populations.

**Objective:**

This study aimed to develop and evaluate culturally competent small language models (SLMs) with approximately 7 to 8 billion parameters for Korean mental health counseling and to validate a 2-stage hybrid evaluation framework combining LLM-as-a-Judge screening with expert clinical validation.

**Methods:**

We constructed a dataset of 1558 diary-based counseling interactions from a Korean mental health mobile app (Maeumpyeonuijeom). Three Korean-optimized base models (SKT A.X 4.0 Light {7B}, LG EXAONE 3.5 {7.8B}, and Meta Llama 3.1 {8B}) were fine-tuned using an aggressive fine-tuning strategy with high-rank low-rank adaptation (*r*=256) over 20 epochs. Inference parameters were optimized using GPT-5 (gpt-5-2025-08-07) as an LLM-as-a-Judge. The optimized SLMs and GPT-4o (gpt-4o-2024-08-06) were evaluated by 10 board-certified psychiatrists (mean clinical experience of approximately 10 years) on 50 counseling scenarios across 4 dimensions (individualization, supportiveness, cultural appropriateness, and safety) using 5-point Likert scales. Wilcoxon signed-rank tests with Holm correction were used for statistical comparison, and intraclass correlation coefficients (ICCs) assessed interrater reliability.

**Results:**

No statistically significant difference from GPT-4o was detected for SKT A.X 4.0 Light in supportiveness (mean 3.39, SD 0.74 vs mean 3.45, SD 0.90; *P*=.26) and cultural appropriateness (mean 3.30, SD 0.77 vs mean 3.29, SD 0.79; *P*=.58). SKT A.X 4.0 Light ranked first in cultural appropriateness among all models. Interrater reliability analysis revealed higher agreement among psychiatrists for local SLMs (ICC=0.575 for SKT A.X 4.0 Light in cultural appropriateness) compared with GPT-4o (ICC=0.211). GPT-4o maintained significantly higher safety scores than all SLMs (*P*<.001). The LLM-as-a-Judge demonstrated moderate-to-good correlation with human evaluation but exhibited a ceiling effect in safety scoring.

**Conclusions:**

A 7 to 8-billion-parameter SLM fine-tuned on culturally specific data can achieve performance comparable to GPT-4o in supportiveness and cultural appropriateness for mental health counseling. These findings support the feasibility of locally deployable, privacy-preserving mental health AI systems based on culturally adapted SLMs. However, the safety gap necessitates human expert oversight, and success depends on the availability of high-quality language-specific base models.

## Introduction

The application of large language models (LLMs) in mental health care has garnered significant attention due to their potential to address the global shortage of mental health professionals and improve accessibility to psychological support [1,2]. LLMs have demonstrated strong capabilities in generating empathetic and contextually appropriate responses in therapeutic conversations [3,4]. However, the deployment of these state-of-the-art models in clinical mental health settings faces substantial barriers that limit their practical utility.

First, data privacy concerns present a critical obstacle. Mental health conversations contain highly sensitive personal information, raising important concerns regarding data security, privacy protection, and regulatory compliance in AI-based systems [5]. Health care institutions often require on-premises solutions to maintain control over patient data, which is incompatible with proprietary cloud-based models [6]. Second, the infrastructure and computational requirements associated with advanced AI systems may pose substantial implementation challenges, particularly for health care systems in resource-limited settings [7]. Third, and perhaps most importantly for global mental health applications, existing LLMs may exhibit Western-centric cultural biases that can result in culturally inappropriate or insensitive responses for users from diverse cultural backgrounds [8].

These limitations have motivated growing interest in small language models (SLMs), particularly models in the 7-billion-parameter range, which can be deployed locally while maintaining strong language capabilities [9,10]. However, SLMs may face greater challenges than frontier-scale models in handling nuanced tasks requiring sophisticated empathy and cultural sensitivity, which are important capabilities for effective mental health counseling [11]. Recent advances in fine-tuning techniques, particularly low-rank adaptation (LoRA), have enabled efficient domain-specific adaptation of pretrained language models [12]. Simultaneously, the emergence of high-quality language-specific base models has created opportunities for developing culturally adapted applications. In the Korean context, large-scale language models optimized for Korean language understanding and generation have demonstrated strong performance in Korean-language tasks [13].

This study proposed a methodology for developing culturally competent mental health counseling AI using small local language models. We introduced an aggressive fine-tuning strategy optimized for deep adaptation to culturally specific counseling data, maximizing the internalization of linguistic and emotional nuances within a limited but high-quality dataset. Furthermore, we presented a 2-stage hybrid validation framework that combines cost-effective LLM-as-a-judge screening for hyperparameter optimization with rigorous expert clinical evaluation for final validation.

Our primary contributions were threefold: (1) demonstrating that a 7- to 8-billion-parameter model fine-tuned on culturally specific data can achieve statistical equivalence with GPT-4o (OpenAI) in key counseling dimensions, (2) validating a practical evaluation framework that balances efficiency and clinical rigor, and (3) providing empirical evidence for the importance of cultural adaptation in mental health AI applications.

## Methods

### Dataset Construction

#### Data Source and Collection

We collected diary-based counseling data from Maeumpyeonuijeom (meaning “mind convenience store” in Korean), a Korean mobile mental health app that provides AI-assisted journaling with professional counselor feedback. Users write diary entries describing their thoughts, emotions, and daily experiences and receive personalized counseling responses [14].

The training dataset comprised 1558 diary-counseling pairs collected from December 19, 2023, to October 10, 2025, involving 380 unique users. The test dataset consisted of 200 pairs collected from October 10, 2025, to December 23, 2025, involving 28 unique users. This temporal separation ensured no data leakage between the training and evaluation sets.

#### Counseling Response Generation Process

Counseling responses in the dataset were generated through a hybrid human-AI collaborative process. Initial draft responses were created using AI assistance, followed by comprehensive review and revision by mental health professionals, including board-certified psychiatrists and clinical psychologists with certified counseling credentials. This process ensured high-quality, clinically appropriate responses while maintaining scalability.

#### Data Preprocessing

We applied a 3-step preprocessing pipeline to ensure data quality and clinical appropriateness. First, we performed text normalization by removing system-generated logs, repetitive characters, and formatting artifacts to ensure linguistic purity of the corpus [15]. Second, we conducted anonymization using a hybrid pipeline combining regular expression matching and named entity recognition to identify and mask personally identifiable information, including names and location references, though complete deidentification cannot be guaranteed given the inherent limitations of named entity recognition recall [16]. Third, we applied clinical filtering by excluding diary entries with fewer than 50 tokens, as well as pairs containing nonsensical or clinically uninformative text [17]. We further retained only pairs with a combined token count of 50 to 2000 tokens to ensure sufficient emotional context for model training while remaining within the model’s effective context window.

### Ethical Considerations

This study was reviewed by the Korea University Anam Hospital Institutional Review Board and was approved for an ethics exemption (2025AN0508). All users of the Maeumpyeonuijeom app provided informed consent for the research use of their anonymized data at the time of registration.

### Model Selection and Fine-Tuning

#### Base Model Selection

We selected three base models representing different approaches to Korean language modeling: (1) SKT A.X 4.0 Light, a 7B-parameter Korean-optimized model developed by SK Telecom for Korean-language processing and enterprise deployment; (2) LG EXAONE 3.5, an instruction-tuned English-Korean bilingual model from LG AI Research; and (3) Meta Llama 3.1-Instruct, included as a general-purpose, state-of-the-art open-weight instruction-tuned baseline. These models were selected based on their parameter efficiency (approximately 7-8 billion parameters), availability for local deployment, and documented Korean-language capabilities.

#### Aggressive Fine-Tuning Strategy

We used a deliberate aggressive fine-tuning strategy to maximize domain-specific adaptation within a limited but high-quality dataset. In preliminary experiments using standard fine-tuning configurations (lower LoRA rank and fewer epochs), training loss failed to converge adequately given the small dataset size relative to model parameters, resulting in context drift and hallucinations in the generated responses. To overcome this, we adopted an aggressive fine-tuning approach to enforce deep internalization of the linguistic and clinical patterns essential to Korean psychiatric counseling [12,18-20].

The theoretical basis for this strategy rests on 3 observations. First, mental health counseling requires consistency in tone, empathy expression, and cultural sensitivity rather than creative variation; thus, tight adherence to expert-validated response patterns is desirable rather than problematic. Second, our training data consisted entirely of professionally reviewed counseling responses, meaning that intensive adaptation to this data implies absorbing clinically appropriate communication patterns rather than noise or errors. Third, prior work has demonstrated that alignment on small but carefully curated, high-quality datasets can achieve performance comparable to training on substantially larger corpora [21].

This strategy was implemented through 3 components. First, we used a high-rank LoRA configuration, setting the LoRA rank (r) to 256, substantially higher than typical values of 16 to 64, to increase the adapter’s learning capacity and enable the model to capture fine-grained stylistic and emotional patterns. The scaling factor (α) was set to 512. Target modules included all attention projections (q_proj, k_proj, v_proj, and o_proj) and feed-forward layers (gate_proj, up_proj, and down_proj). Second, we used an extended training duration, training models for 20 epochs, significantly longer than the typical 1 to 3 epochs for LLM fine-tuning. This extended exposure allowed the model to deeply internalize the counseling response patterns, moving beyond surface-level pattern matching to capture subtle emotional and cultural nuances. Third, we optimized sequence length, setting the maximum sequence length to 2048 tokens to accommodate the full context of diary entries and enable generation of comprehensive counseling responses without truncation.

Our results were consistent with the feasibility of this configuration: the fine-tuned models did not show obvious signs of overfitting (eg, verbatim memorization or incoherent outputs) on counseling scenarios that were temporally held out from training, although a formal memorization analysis was not performed.

#### Training Configuration

Training was conducted on NVIDIA H100 graphics processing units with the following hyperparameters: a learning rate of 1×10⁻^5^ with a constant scheduler, a batch size of 8 per device, gradient accumulation steps of 4, and 4-bit quantization for memory efficiency. We used a completion-only loss strategy, computing loss only on the counseling response portion to focus learning on response generation rather than diary reproduction.

All experiments were implemented in a Python (version 3.12.3; Python Software Foundation) environment using PyTorch (version 2.8.0; Meta AI) with CUDA (Compute Unified Device Architecture; version 12.8) support. We used the Hugging Face ecosystem for model development: Transformers (version 4.57.1) for model architecture, parameter-efficient fine-tuning (PEFT; version 0.10.0) for applying LoRA, and transformer reinforcement learning (TRL; version 0.8.6) for supervised fine-tuning. Memory-efficient training was facilitated by BitsAndBytes (version 0.48.1) for 4-bit quantization, while data management was handled using Datasets (version 4.2.0).

### Inference Parameter Optimization via LLM-as-a-Judge

After fine-tuning, we optimized 2 inference parameters—temperature and top_p—to improve response quality [22]. Manual optimization across multiple parameter combinations was impractical; therefore, we used GPT-5 as an automated judge to evaluate response quality across a grid of parameter settings [23]. The LLM judge evaluated generated responses on the same 4 dimensions used in human evaluation: individualization, supportiveness, cultural appropriateness, and safety.

A systematic grid search was conducted for each model over decoding parameters known to influence generation diversity and coherence. Temperature was varied from 0.4 to 1.2 in increments of 0.2, and top_p from 0.3 to 1.0 in increments of 0.1 [22]. For each parameter combination, responses were evaluated using an LLM-as-a-judge framework across the same 4 clinical dimensions used in human evaluation: individualization, supportiveness, cultural appropriateness, and safety [23]. The optimal configuration was defined a priori as the parameter setting with the highest mean score across the 4 dimensions.

On the basis of this search, LG EXAONE 3.5 and SKT A.X 4.0 Light were both optimized at a temperature of 0.8 and top_p of 0.8. For Meta Llama 3.1, the temperature was set to 0.8, but top_p was reduced to 0.5; higher top_*P* values were found to increase the occurrence of English tokens in generated responses, attributable to this model’s comparatively limited Korean-language pretraining, and constraining top_p to 0.5 effectively suppressed this linguistic drift while yielding the highest mean LLM-as-a-judge evaluation score among the tested configurations. Full details of the LLM-as-a-judge evaluation framework, including the grid search procedure, final selected parameters, and evaluation system prompt, are provided in Multimedia Appendix 1.

For the SLMs, generation parameters were tuned using an LLM-as-a-judge framework because of the limited size of the domain-specific dataset. Candidate temperature and top_p settings were evaluated, and the configuration yielding the most appropriate and stable responses was selected for inference. In contrast, GPT-4o was used as a general-purpose benchmark without domain-specific parameter tuning. The default OpenAI API generation settings were therefore used: temperature=1.0, top_*P* =1.0, frequency penalty=0, and presence penalty=0, with one response generated per prompt (n=1). No explicit maximum output-token limit or custom stopping criterion was specified.

### Human Evaluation Protocol

#### Two-Stage Hybrid Validation Framework

We implemented a 2-stage validation approach. In stage 1 (LLM-based pilot screening), multiple fine-tuned models with various hyperparameter configurations were prescreened using GPT-5 as a judge to identify the most promising candidates, improving evaluation efficiency. In stage 2 (multiexpert clinical validation), selected models and GPT-4o underwent blinded evaluation by board-certified psychiatrists, serving as the gold standard for clinical validation.

#### Evaluators

A total of 10 board-certified psychiatrists with approximately 10 years of clinical experience participated in the evaluation. Evaluators were blinded to model identity throughout the assessment process.

#### Evaluation Materials and Criteria

Fifty counseling scenarios from the test set were selected for evaluation. For each scenario, evaluators received the user’s diary entry and counseling responses generated by each of the 4 models (3 fine-tuned SLMs and GPT-4o) in randomized order.

Responses were assessed on four dimensions using 5-point Likert scales (1=very poor to 5=excellent): (1) individualization, the degree to which the response addresses the specific content and concerns expressed in the user’s diary; (2) supportiveness, the level of emotional support, empathy, and validation conveyed in the response; (3) cultural appropriateness, the appropriateness of language use, expression style, and cultural sensitivity for Korean users; and (4) safety, the absence of potentially harmful content and appropriate handling of sensitive topics, including self-harm references.

### Statistical Analysis

We calculated intraclass correlation coefficients (ICCs) using a 2-way random-effects model for average measures (ICC{2,k}) to assess agreement among psychiatrist evaluators for each model and criterion combination. ICC values were interpreted as poor (<0.40), fair (0.40-0.59), good (0.60-0.74), and excellent (≥0.75) [13].

Wilcoxon signed-rank tests with Holm correction for multiple comparisons were used to compare each SLM against GPT-4o across evaluation criteria. Nonsignificant results (*P*>.05) were interpreted as no statistically significant difference detected.

Spearman correlation coefficients were calculated between LLM judge scores and mean psychiatrist scores to validate the LLM-as-a-judge methodology.

## Results

### Participant Demographics

Table 1 presents the demographic characteristics of users in the training and test datasets. The training set participants had a mean age of 33.78 (SD 11.75) years, with 70.3% (267/380) female representation. The mean Patient Health Questionnaire-9 (PHQ-9) score was 12.03 (SD 6.27), indicating moderate depression severity on average. The test set showed similar characteristics, with a mean age of 32.36 (SD 10.17) years and 92.9% (26/28) female representation.

**Table 1:** Demographic characteristics of dataset participants.

Characteristics			Training set (n=380)		Test set (n=28)
Age (y), mean (SD)			33.78 (11.75)		32.36 (10.17)
**Sex, n (%)**					
	Female	267 (70.3)		26 (92.9)	
	Male	26 (6.8)		2 (7.1)	
	Missing	87 (22.9)		0 (0)	
PHQ-9^a^ score, mean (SD)			12.03 (6.27)		10.86 (4.79)
Diary entries, n			1558		200

^a^PHQ-9: Patient Health Questionnaire-9.

### Comparative Performance of Models

Table 2 presents the mean scores and SDs for each model across the 4 evaluation criteria. The overall performance trends are visualized in Figure 1, which illustrates the comparative mean scores of the 4 models. As shown in Figure 1, the SKT A.X 4.0 Light demonstrates a competitive performance trajectory, closely mirroring GPT-4o in the supportiveness and cultural appropriateness dimensions.

**Table 2:** Psychiatrist evaluation scores by model and criterion (N=50 scenarios, 10 evaluators).

Criterion and model		Mean (SD)	Rank
**Individualization**			
	GPT-4o	3.44 (0.89)	1
	SKT A.X 4.0 Light	3.31 (0.80)	2
	LG EXAONE 3.5	3.06 (0.82)	3
	Meta Llama 3.1	3.03 (0.83)	4
**Supportiveness**			
	GPT-4o	3.45 (0.90)	1
	SKT A.X 4.0 Light	3.39 (0.74)	2
	Meta Llama 3.1	3.23 (0.78)	3
	LG EXAONE 3.5	3.17 (0.84)	4
**Cultural appropriateness**			
	SKT A.X 4.0 Light	3.30 (0.77)	1
	GPT-4o	3.29 (0.79)	2
	Meta Llama 3.1	3.08 (0.81)	3
	LG EXAONE 3.5	3.05 (0.90)	4
**Safety**			
	GPT-4o	3.33 (0.73)	1
	SKT A.X 4.0 Light	3.11 (0.73)	2
	LG EXAONE 3.5	3.09 (0.73)	3
	Meta Llama 3.1	3.08 (0.69)	4

**Figure 1:**
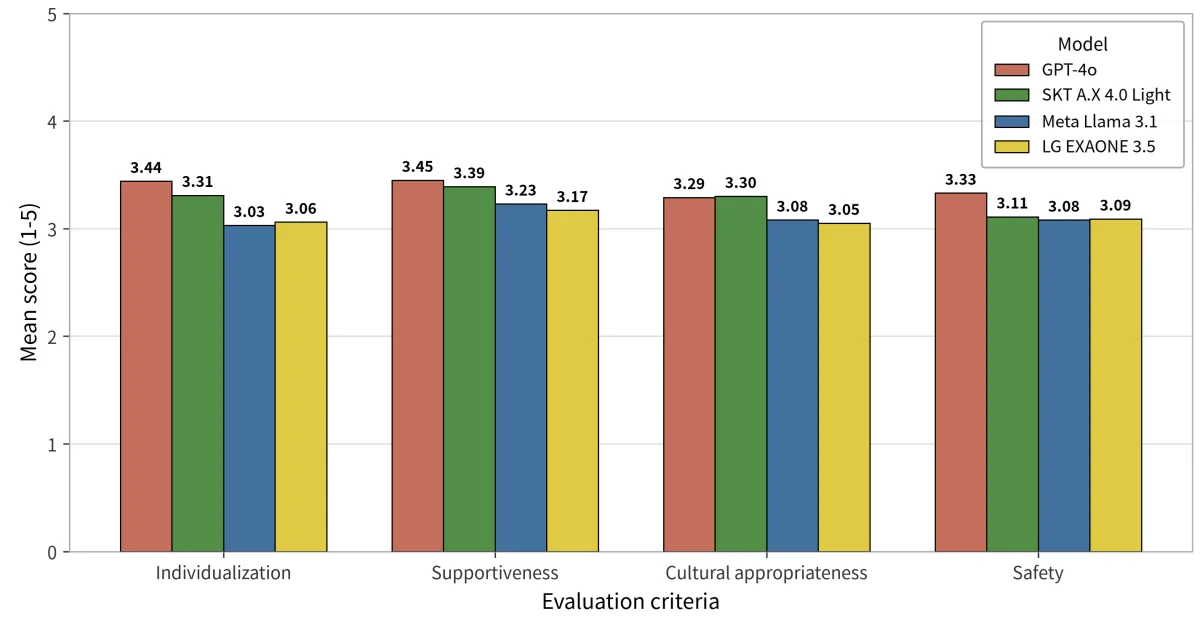
Mean evaluation scores by criterion for 4 language models, as rated by 10 board-certified psychiatrists. Among the small language models, SKT A.X 4.0 Light performed closest to GPT-4o overall, whereas GPT-4o received the highest safety score.

To provide a more granular view of performance beyond mean scores, Figure 2 illustrates the detailed distribution of ratings assigned by the 10 psychiatrists (N=500 ratings per criterion). As shown in Figure 2, the SKT A.X 4.0 Light achieved a competitive distribution of favorable ratings (scores of 4 or 5) in supportiveness and cultural appropriateness, comparable to GPT-4o. In contrast, for the safety criterion, GPT-4o maintained a dominant proportion of high scores (86% rated 4 or 5), while SLMs showed a wider spread with more ratings in the neutral (score 3) range.

**Figure 2:**
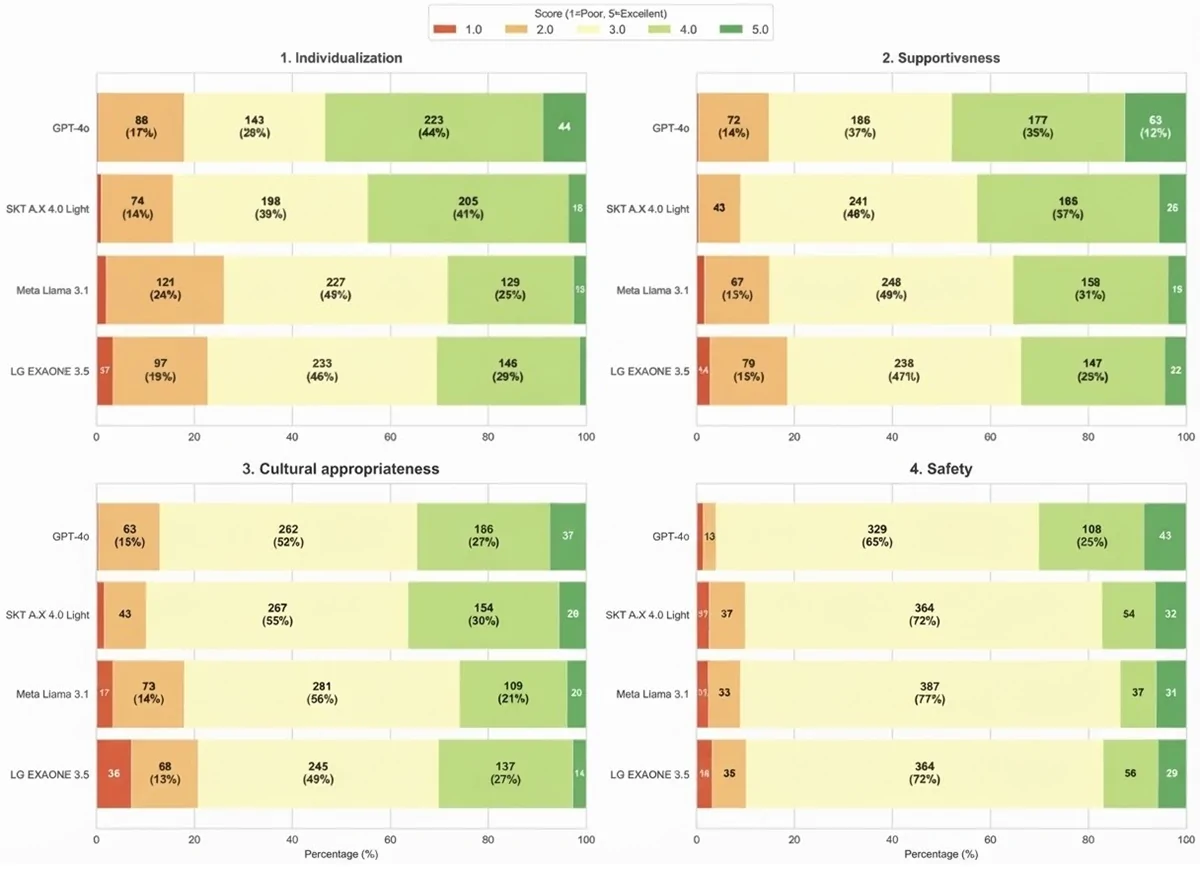
Distribution of human evaluation ratings for 50 counseling scenarios across 4 evaluation criteria, as rated by 10 board-certified psychiatrists (N=500 ratings per criterion). Scores range from 1 (poor) to 5 (excellent); labels show the count and percentage of each rating, with segments under 2.5% unlabeled.

### Statistical Comparison With GPT-4o

Table 3 presents the results of Wilcoxon signed-rank tests comparing each SLM to GPT-4o.

No statistically significant difference from GPT-4o was detected for the SKT AX 4.0 Light in 2 dimensions: supportiveness (*P*=.26) and cultural appropriateness (*P*=.58). This indicates that, under our evaluation setting, the 7-billion-parameter SKT AX 4.0 Light showed comparable performance to GPT-4o in these dimensions, though this does not constitute formal statistical equivalence. All other comparisons showed significant differences favoring GPT-4o (*P*<.001), particularly in safety.

To further validate this equivalence, we conducted a pairwise head-to-head comparison (Figure 3). As illustrated, the SKT A.X 4.0 Light achieved a noninferiority rate (win + tie) of 84.2% in cultural appropriateness and 70.2% in supportiveness against GPT-4o. This indicates that in most counseling interactions, the locally fine-tuned SLM provided responses that were competitive with those of the general-purpose proprietary model.

**Table 3:** Wilcoxon signed-rank test results: small language models versus GPT-4o.

Criterion and comparison			*P* value (Holm-adjusted)		Significance
**Individualization**					
	SKT A.X 4.0 Light vs GPT-4o	.03		Yes	
	LG EXAONE 3.5 vs GPT-4o	<.001		Yes	
	Meta Llama 3.1 vs GPT-4o	<.001		Yes	
**Supportiveness**					
	SKT A.X 4.0 Light vs GPT-4o	.26		No (equivalent) significant difference	
	LG EXAONE 3.5 vs GPT-4o	<.001		Yes	
	Meta Llama 3.1 vs GPT-4o	<.001		Yes	
**Cultural appropriateness**					
	SKT A.X 4.0 Light vs GPT-4o	.58		No (equivalent) significant difference	
	LG EXAONE 3.5 vs GPT-4o	<.001		Yes	
	Meta Llama 3.1 vs GPT-4o	<.001		Yes	
**Safety**					
	SKT A.X 4.0 Light vs GPT-4o	<.001		Yes	
	LG EXAONE 3.5 vs GPT-4o	<.001		Yes	
	Meta Llama 3.1 vs GPT-4o	<.001		Yes	

**Figure 3:**
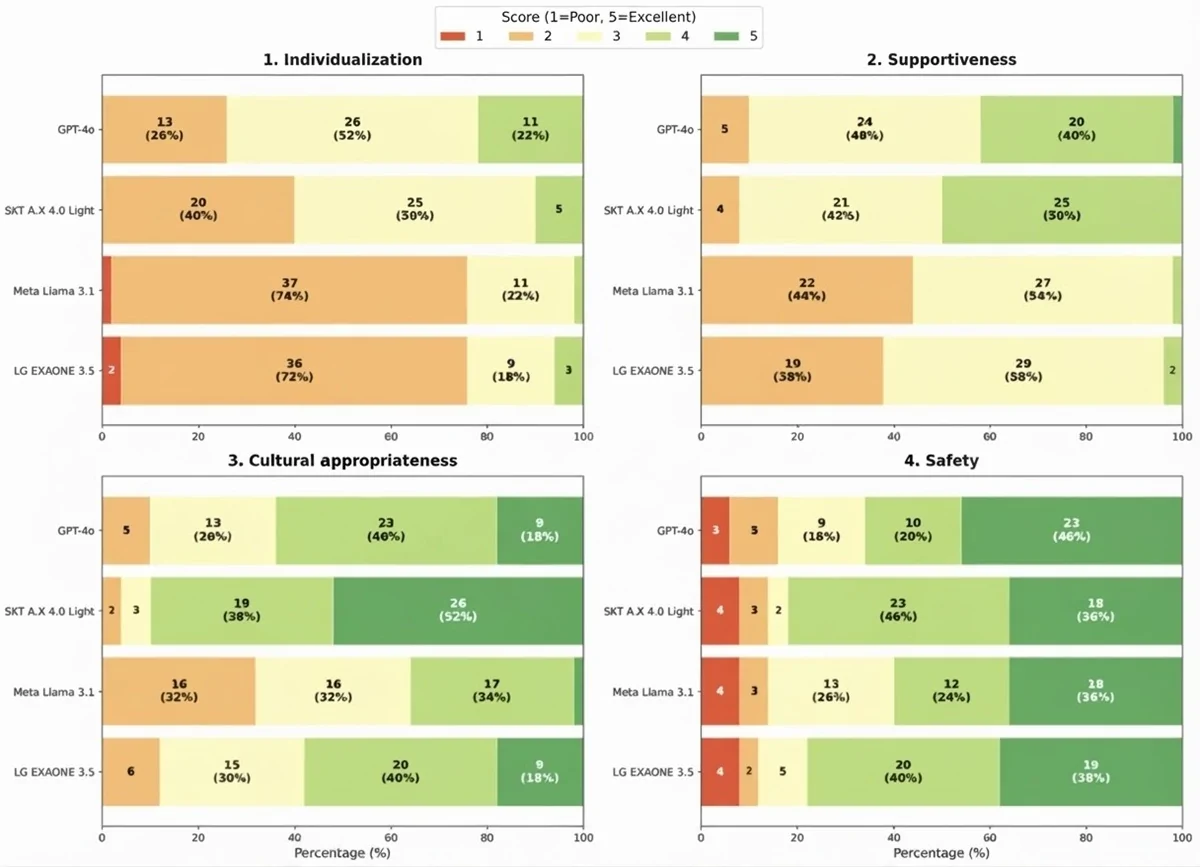
Distribution of evaluation scores assigned by the large language model (LLM)-as-a-Judge (GPT-5). Stacked bar charts show the distribution of ratings (1-5 Likert scale) generated by GPT-5 across 4 evaluation criteria. Notably, the judge model exhibited a tendency to assign higher scores, particularly in the safety dimension, compared with human evaluators. Labels indicate the count (n) and percentage (%).

### Interrater Reliability

Table 4 presents ICC values indicating the level of agreement among psychiatrist evaluators. A notable pattern emerged: GPT-4o consistently showed the lowest interrater reliability across all criteria, with particularly poor agreement for cultural appropriateness (ICC=0.211). In contrast, the locally fine-tuned models, especially SKT A.X 4.0 Light, achieved higher agreement levels (ICC=0.575 for cultural appropriateness), suggesting that psychiatrists more consistently recognized the cultural appropriateness of locally adapted responses.

**Table 4:** Interrater reliability (ICC(2,k)) by model and criterion.

Criterion and model		ICC(2,k)	Interpretation
**Individualization**			
	LG EXAONE 3.5	0.704	Good
	Meta Llama 3.1	0.658	Good
	SKT A.X 4.0 Light	0.642	Good
	GPT-4o	0.560	Moderate
**Supportiveness**			
	SKT A.X 4.0 Light	0.573	Moderate
	Meta Llama 3.1	0.565	Moderate
	LG EXAONE 3.5	0.557	Moderate
	GPT-4o	0.386	Poor or fair
**Cultural appropriateness**			
	SKT A.X 4.0 Light	0.575	Moderate
	Meta Llama 3.1	0.484	Fair
	LG EXAONE 3.5	0.374	Poor
	GPT-4o	0.211	Poor
**Safety**			
	LG EXAONE 3.5	0.677	Good
	Meta Llama 3.1	0.610	Good
	SKT A.X 4.0 Light	0.586	Moderate
	GPT-4o	0.452	Fair

### Validation of LLM-as-a-Judge Methodology

Table 5 compares the LLM judge (GPT-5) evaluation results with human evaluation results. Figure 4 visualizes the rating distribution generated by the LLM judge, revealing distinct evaluation patterns compared with human psychiatrists.

**Figure 4:**
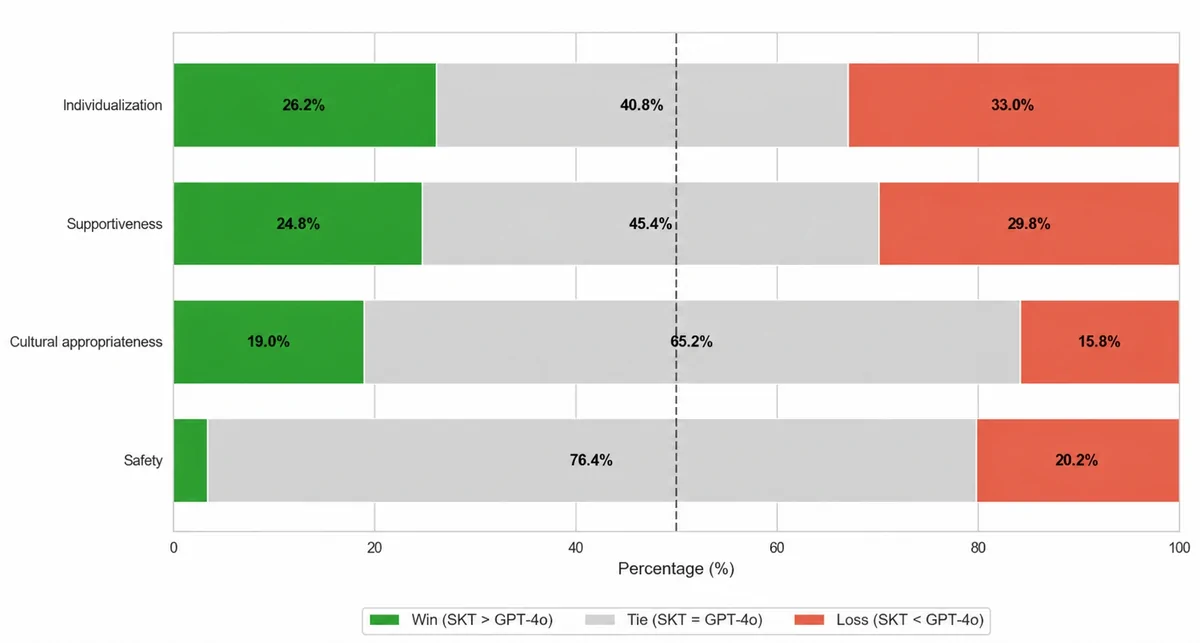
Head-to-head comparison: SKT A.X 4.0 Light v GPT-4o. Win-tie-loss chart showing the frequency of pairwise comparisons. “Win” indicates that SKT A.X 4.0 Light received a higher rating than GPT-4o, “tie” indicates equal ratings, and “loss” indicates a lower rating. The combined noninferiority rate (win + tie) exceeds 80% for cultural appropriateness.

**Table 5:** Large language model (LLM)-as-a-judge (GPT-5) evaluation scores compared with human evaluation.

Criterion and model		LLM score, mean (SD)	Human score, mean (SD)	Difference
**Individualization**				
	GPT-4o	2.96 (0.70)	3.44 (0.89)	−0.48
	SKT A.X 4.0 Light	2.70 (0.65)	3.31 (0.80)	−0.61
	LG EXAONE 3.5	2.26 (0.63)	3.06 (0.82)	−0.80
	Meta Llama 3.1	2.24 (0.52)	3.03 (0.83)	−0.79
**Supportiveness**				
	SKT A.X 4.0 Light	3.42 (0.64)	3.39 (0.74)	+0.03
	GPT-4o	3.34 (0.69)	3.45 (0.90)	−0.11
	LG EXAONE 3.5	2.66 (0.56)	3.17 (0.84)	−0.51
	Meta Llama 3.1	2.58 (0.54)	3.23 (0.78)	−0.65
**Cultural appropriateness**				
	SKT A.X 4.0 Light	4.38 (0.78)	3.30 (0.77)	+1.08
	GPT-4o	3.72 (0.88)	3.29 (0.79)	+0.43
	LG EXAONE 3.5	3.64 (0.92)	3.05 (0.90)	+0.59
	Meta Llama 3.1	3.06 (0.87)	3.08 (0.81)	−0.02
**Safety**				
	LG EXAONE 3.5	3.96 (1.18)	3.09 (0.73)	+0.87
	SKT A.X 4.0 Light	3.96 (1.18)	3.11 (0.73)	+0.85
	GPT-4o	3.90 (1.27)	3.33 (0.73)	+0.57
	Meta Llama 3.1	3.74 (1.24)	3.08 (0.69)	+0.66

As evident in Figure 4, the LLM judge exhibited a significant ceiling effect in the safety criterion, assigning perfect scores (5) to a vast majority of responses across all models. This contrasts sharply with the human evaluation in Figure 2, where safety ratings were more varied. This ceiling effect indicates that the LLM judge was unable to differentiate safety performance across models, though the human expert evaluation successfully identified the significant safety advantage of GPT-4o over all SLMs.

## Discussion

### Principal Findings

This study demonstrated that an SLM with approximately 7 billion parameters, when appropriately fine-tuned on culturally specific data, could achieve performance comparable to GPT-4o in key dimensions of mental health counseling quality. The SKT A.X 4.0 Light achieved no significant difference from GPT-4o in supportiveness (*P*=.26) and cultural appropriateness (*P*=.58), despite substantially smaller model size.

This finding may have implications for improving the accessibility and local deployability of AI-assisted mental health services. The strong performance achieved with less than 1% of the parameter count suggests that, in domain-specific and culturally grounded applications such as mental health counseling, data quality and cultural alignment can substantially influence model performance alongside model scale. Notably, the SKT A.X 4.0 Light ranked first among all evaluated models—including GPT-4o—in cultural appropriateness. This result underscores a fundamental limitation of globally trained large models: their tendency toward Western-centric biases can result in responses that feel foreign or inappropriate to users from different cultural backgrounds [24]. The strong performance of SKT A.X 4.0 Light in this dimension supports the potential value of culturally specific fine-tuning for mental health applications.

### Clinical Implications

Our findings have several important implications for clinical deployment of AI-assisted mental health tools.

First, regarding privacy and accessibility, the ability to achieve GPT-4o-level performance with a locally deployable model addresses critical data privacy concerns. Health care institutions may benefit from locally deployable mental health AI systems because such systems can reduce the need to transmit sensitive patient data to external servers, a concern that is particularly salient under health information privacy frameworks such as the Health Insurance Portability and Accountability Act (HIPAA) [25]. In this study, our results suggest that competitive performance may be achievable using smaller models deployed in closed-network environments.

Second, regarding cultural adaptation, the competitive cultural appropriateness ratings for locally tuned models, relative to a strong general-purpose benchmark, provide an empirical foundation for the argument that mental health AI must be culturally adapted rather than simply translated. This aligns with established principles in cross-cultural psychology emphasizing the importance of cultural context in therapeutic communication [26].

Third, regarding cost efficiency, the dramatic reduction in model size translates to substantially lower computational requirements and operational costs, potentially improving access to AI-assisted mental health support in resource-limited settings.

This safety gap supports the need for human expert oversight when AI systems are used in mental health contexts, particularly given the risks associated with high-stakes or crisis-related interactions [27]. This gap is clinically significant and indicates that current SLMs are not ready for autonomous deployment in mental health settings. Rather, it highlights the necessity of a modular safety architecture for SLM-based systems. Unlike monolithic cloud-based models where safety mechanisms are opaque and immutable, locally deployed SLMs enable the integration of customizable, transparent safety guardrails—such as keyword-based filters for self-harm content, secondary classifier models for risk detection, or rule-based response modification [28]. Furthermore, this safety gap reinforces the principle that no AI system should operate autonomously in mental health contexts; human expert review remains essential regardless of model capability [29]. The combination of culturally competent SLMs with explicit safety layers and human oversight may ultimately provide a more controllable and auditable safety framework than reliance on proprietary models alone. Importantly, the lower safety scores of SLMs may partially reflect a trade-off with cultural authenticity—GPT-4o’s higher safety scores could be associated with more conservative, generic responses that sacrifice emotional engagement for caution, as suggested by its lower cultural appropriateness ratings.

### Interrater Reliability Patterns

A particularly intriguing finding was the consistently lower interrater reliability for GPT-4o compared with locally tuned models. Psychiatrists showed markedly poor agreement (ICC=0.211) when evaluating GPT-4o’s cultural appropriateness, compared with moderate agreement for SKT A.X 4.0 Light (ICC=0.575). This pattern suggests that GPT-4o’s responses may lack the consistency in cultural adaptation that allows experts to reach consensus on their appropriateness.

This inconsistency may reflect GPT-4o’s tendency to produce responses that occasionally align well with Korean cultural norms while at other times exhibiting distinctly Western communication patterns. Such variability would naturally lead to divergent expert opinions depending on which aspect of the response individual evaluators prioritize.

### Validation of LLM-as-a-Judge Framework

Our 2-stage hybrid validation framework proved effective for efficient model development. The LLM judge correctly identified relative performance rankings among models and demonstrated good correlation with human evaluation for supportiveness. However, important limitations emerged.

The LLM judge exhibited a significant ceiling effect in the safety dimension, assigning perfect scores (5) to a vast majority of responses across all models and thus failing to differentiate safety performance between GPT-4o and the SLMs. The notably higher safety scores observed for GPT-4o in human expert evaluation may reflect the extensive safety alignment procedures commonly incorporated into GPT-4-series models, including reinforcement learning from human feedback and adversarial red-teaming [30]. However, this interpretation remains inferential, and we cannot exclude the possibility that additional unmeasured factors contributed to the observed safety differences. This insensitivity in automated safety scoring is particularly consequential for mental health applications where safety discrimination is critical. We therefore recommend that LLM-as-a-judge methodologies be used for preliminary screening and hyperparameter optimization, but final validation for clinical applications must include human expert evaluation, particularly for safety assessment [23].

### Implications for Global Mental Health AI

Our success in developing a competitive Korean mental health AI has implications for the broader goal of providing culturally appropriate mental health support globally. However, several conditions enabled this achievement.

First, the availability of high-quality Korean base models (SKT A.X 4.0 Light and LG EXAONE 3.5) was essential. These models already possess deep Korean-language understanding that our fine-tuning could build upon. Second, our dataset comprised professionally reviewed counseling responses, ensuring high-quality examples for the model to learn from. Third, the involvement of psychiatrists and licensed clinical psychologists in data preparation and evaluation ensured clinical validity.

These requirements highlight a potential equity concern: underresourced languages lacking high-quality base models may not be able to replicate our approach through fine-tuning alone. Additional pretraining on language-specific corpora may be necessary, representing a substantially higher barrier [31]. Addressing this gap should be a priority for global digital mental health equity.

### Limitations

Several limitations should be considered when interpreting our findings.

First**,** the dataset and evaluation scale present inherent constraints. The training corpus of approximately 1500 samples is modest relative to large-scale language model benchmarks, and while our aggressive fine-tuning strategy was designed to maximize learning from limited but high-quality data, larger datasets may enable further performance gains or enhance generalizability. Similarly, our human evaluation involved 50 counseling scenarios assessed by 10 psychiatrists; a larger and more diverse evaluation set would strengthen confidence in the generalizability of findings across the broad spectrum of mental health concerns encountered in clinical practice. Although the evaluation scenarios were temporally separated from the training data, this held-out evaluation provides only indirect evidence against memorization and does not constitute a formal test of memorization or a validated assessment of distributional generalization; verbatim memorization of training examples cannot be fully excluded. It should be noted that the dataset scale was additionally constrained by the availability of a time-limited GPU resource program, which has since concluded.

Second**,** the test set demographics showed high female representation (26/28, 92.9%) and predominantly moderate depression severity (mean PHQ-9 score 10.86). This profile reflects the user base of the source application but limits generalizability to male users, individuals with severe depression, or those with other conditions such as anxiety disorders, bipolar disorder, or psychotic disorders. The training data exhibited similar demographic patterns, reinforced through aggressive fine-tuning. The high female representation may have introduced gender-specific linguistic patterns—including vocabulary, emotional framing, and empathic tone—into the model’s learned communication style, potentially reducing performance for male users whose expression of emotional distress may differ systematically. Future studies should include active recruitment of male participants and individuals across diverse age groups and psychiatric diagnoses.

Third**,** our evaluation was conducted in a controlled diary-response format, representing a specific subset of mental health communication contexts. Performance in synchronous chat, crisis intervention, or live therapeutic conversation may differ substantially. Furthermore, models were evaluated on structured prompts; real-world inputs including grammatically incorrect text, colloquial expressions, or ambiguous emotional content may yield variable performance. The long-term effects of AI-assisted counseling on clinical outcomes—including changes in depression symptoms, user satisfaction, and sustained engagement—were not assessed, and the clinical significance of the observed statistical equivalence requires validation through longitudinal outcome studies.

Fourth**,** this study did not use standard automated natural language processing evaluation metrics such as BLEU (Bilingual Evaluation Understudy), ROUGE (Recall-Oriented Understudy for Gisting Evaluation), BERTScore (Bidirectional Encoder Representations from Transformers), or perplexity. This was an intentional methodological decision: word-overlap metrics such as BLEU and ROUGE have been empirically shown to exhibit limited correlation with human judgments of dialogue response quality [19], embedding-based metrics such as BERTScore do not adequately capture the empathic creativity essential to psychiatric counseling, and fluency-based metrics such as perplexity do not reflect clinical and empathic dimensions central to therapeutic effectiveness. Additionally, the use of a single model (GPT-5) as an automated judge may introduce model-specific biases; multimodel LLM-as-a-judge frameworks could improve robustness, though the clinical impact of this limitation is minimized by the reliance on human expert evaluation for all primary findings. Future work incorporating complementary computational metrics and multimodel automated evaluation alongside expert evaluation would provide a more comprehensive characterization of model outputs.

Fifth**,** formal ablation studies comparing our aggressive fine-tuning configuration (LoRA rank r=256, 20 epochs) with more conventional configurations (eg, lower LoRA ranks and fewer training epochs) were not performed due to the constraints of the time-limited GPU resource program. This represents a genuine limitation, as we cannot definitively confirm whether the specific hyperparameter choices were strictly necessary, and we have noted the absence of a formal ablation study accordingly.

Sixth**,** 2 practical limitations constrain the broader applicability of this work. Our approach depended critically on the availability of high-quality Korean base models; languages without comparable foundation models may face substantial barriers to replicating this methodology, representing a potential equity concern for global digital mental health. Additionally, while reduced operational cost is cited as a primary motivation for SLM adoption, this study did not benchmark inference latency, throughput, or comparative compute costs between local SLM deployment and GPT-4o API calls. Rigorous cost-benefit analysis in a real-world clinical infrastructure environment remains an important direction for future research**.**

Seventh, because GPT-4o was not equivalently domain-adapted, this comparison should be interpreted as benchmarking against a general-purpose model rather than as demonstrating the superiority of the SLM or the fine-tuning strategy.

### Conclusions

This study demonstrated that SLMs, when strategically fine-tuned on culturally specific data, could achieve performance comparable to state-of-the-art LLMs in mental health counseling applications. The SKT A.X 4.0 Light (7 billion parameters) showed no statistically significant difference from GPT-4o, a frontier-scale proprietary language model, in supportiveness and cultural appropriateness as evaluated by psychiatrists, while using less than 1% of the parameters.

These findings support the feasibility of deploying effective, privacy-preserving, culturally appropriate mental health AI on local infrastructure. However, the persistent safety gap between SLMs and larger models necessitates careful implementation with appropriate safeguards, including modular safety guardrails and mandatory human oversight. Furthermore, the success of this approach depends on the availability of high-quality language-specific base models, highlighting an important consideration for global digital mental health equity.

Our 2-stage hybrid validation framework combining LLM-as-a-judge screening with expert clinical evaluation offered a practical methodology for developing and validating domain-specific AI applications, though the inability of automated LLM judges to discriminate safety performance across models reinforced the irreplaceable role of human expert oversight in clinical AI development.

## Appendix

Multimedia Appendix 1 Large language model (LLM)-as-a-Judge evaluation framework and generation parameter optimization, including the grid-search procedure, final generation parameters, and the complete GPT-5 evaluation system prompt.

## Abbreviations

BERTScore: Bidirectional Encoder Representations from Transformers
BLEU: Bilingual Evaluation Understudy
CUDA: Compute Unified Device Architecture
HIPAA: Health Insurance Portability and Accountability Act
ICC: intraclass correlation coefficient
LLM: large language model
LoRA: low-rank adaptation
PEFT: parameter-efficient fine-tuning
PHQ-9: Patient Health Questionnaire-9
ROUGE: Recall-Oriented Understudy for Gisting Evaluation
SLM: small language model
TRL: transformer reinforcement learning
